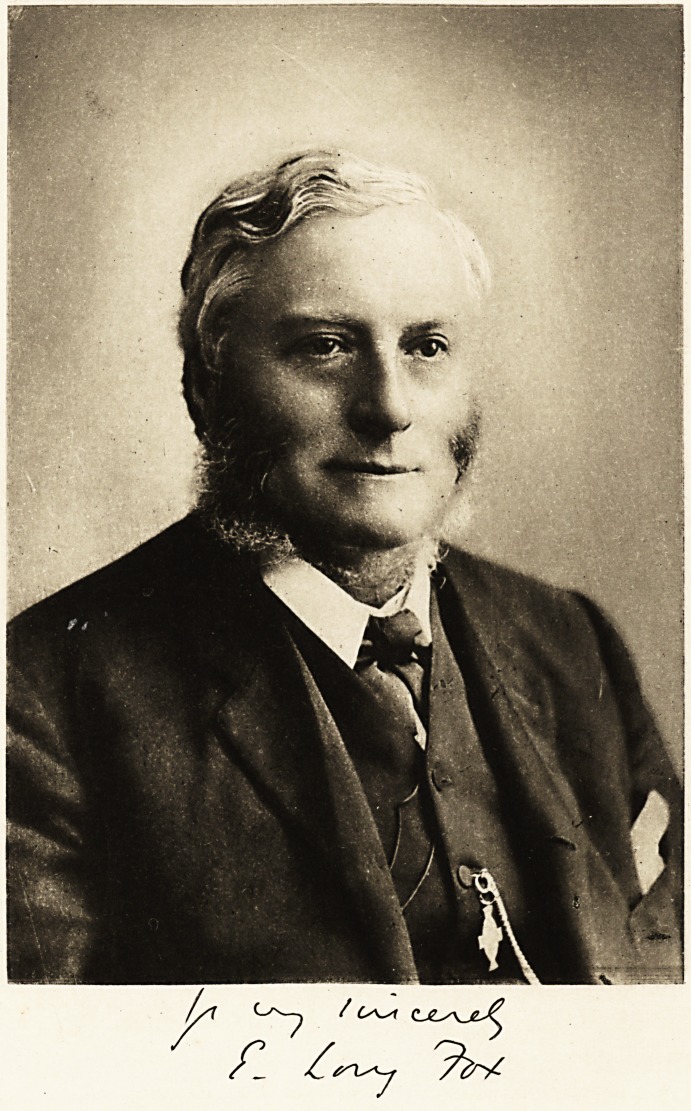# Edward Long Fox

**Published:** 1902-06

**Authors:** 


### The Bristol Medico-Chirurgical Journal.

"*Scire est nescire, nisi id me*
*Scire alius sciret.*"

JUNE, 1902.

### EDWARD LONG FOX, M.D. (Oxon.), F.R.C.P.

Although the death of Dr. Edward Long Fox has not come to us as a surprise, it is none the less a source of keen regret that his very protracted illness should have terminated fatally on Friday, March 28th, 1902. It was a matter of general knowledge that his health had been failing for many months, and his medical friends knew that he had been struggling against gout and glycosuria for many years. When, therefore, it was understood that he was prostrated with neuritis it was from the first feared that his life's work was ended. Such has been the case, and we have now to review that life's work, and gathering what instruction we can from his precept and example "go and do likewise."

He was the eldest son of the late Dr. Francis Ker Fox, of Brislington House. He was born in 1832. After a year or two with a private tutor at Brislington, Edward was sent with his brother Francis to the Bath Grammar School in 1844, where they were both under the discipline of a very rough, unfeeling, and very severe master, who was much dreaded and hated by the boys. Both were sent to Shrewsbury School in 1845, and here they were under the care of the Rev. Benjamin Kennedy, D.D., who was then Head-master. Edward remained there until 1850, and took an active share in all the games of the school and was coxswain of the school eight-oar boat on the river Severn. In 1850 he went to Balliol College, Oxford, of which Dr. Jenkins was then Master, and here he came under the influence of the late Dr. Benjamin Jowett, who was his tutor and who afterwards became Master of Balliol. Here, too, he became pupil and friend of the late well-known Regius Professor, Sir Henry Acland, with whom in later years he was associated as Examiner in Medicine to the University. After obtaining a First Class in the Natural Science Tripos in 1853 he commenced his medical studies in Edinburgh; but he did not remain there long, for he was soon transferred to London, and in 1854 was studying at St. George's Hospital, where he acted as clinical clerk to Dr. Bence Jones. He also attended at the Great Ormond Street Hospital, working especially under Dr. Charles West, and the Brompton Hospital for Consumption, where the late Sir Andrew Clark was one of his most intimate companions. He took the degree of M.B. in 1857, and of M.D. in 1861. Shortly after graduation he was appointed Physician to the Bristol Royal Infirmary, and here many generations of students from 1857 to 1877 will well remember how faithfully all his duties were performed. The name of Fox has been associated with the history of the Infirmary since the year 1786, when his grandfather, Edward Long Fox, held office as Physician for thirty years, and his uncle, Dr. Henry Hawes Fox, was one of the Physicians from 1816 onwards to 1829.

An old pupil writes of him at this time as follows:—

"I have a most pleasing recollection of the day on which I formed one of the small group now living who accompanied him in his first round through the wards of the Bristol Royal Infirmary, and can well recall the favourable impression which he made on us all on that memorable occasion. How tenderly he examined all his poor patients. We were all greatly struck with his humble and characteristic remarks to us on that his first visit. Turning to us, he said: 'I wish to say that as I have only just passed out of the student stage myself, I shall feel greatly pleased should any of you notice anything overlooked in my walk and practice here that might be of importance in the treatment of cases, if you would kindly remind me of the fact; for by such means we shall be serving the patients as well as helping one another.' Such a remark took us all by surprise, and from that day to this the impression of the kind of man he was never left our memories or our hearts."

An old colleague and friend of forty years in a neighbouring city writes as follows :—

"I sought his assistance now and then about difficult cases. His logical alertness made me feel sometimes as if he were too quick in his work: his mental operations were so rapid that I could not always follow them. But his professional judgment was sure and almost unerring. That he was an optimist in prognosis may have been a 'swing' from the pessimist bias of John Addington Symonds.

"After all, character is the great thing. You may be indeed proud of Edward Long Fox as a fellow-citizen: his example and influence were always 'on the better side'; he was magnificently generous, and his hospitality was a proverb everywhere. As a Churchman he was loyal and true. Taking him altogether, he ought to be a precious memory to the inhabitants of Bristol."

The following reminiscences from the pen of one of his old pupils and clinical clerks (Dr. Lionel A. Weatherly) cannot fail to be of interest :—

"When in 1868 I was apprenticed to my father I well recollect Dr. E. L. Fox coming down to meet him in consultation. His anticipated arrival filled me with a certain amount of awe; but within a few moments of his entering the house his kindly interest in me and his encouraging words made me feel that I had found a real friend. When in 1869 my father decided to enter me under Dr. E. Long Fox at the Bristol Royal Infirmary I was indeed delighted, and never have I regretted my good fortune in having been clinical clerk to such a sympathetic and ever-observant physician. Those student days will long be remembered, and those happy strawberry parties which he gave and the kindly word of welcome he had for us all. Truly he was the student's good friend, and many are the kind actions known only to himself and to those who in their trouble sought his ever-wise advice.

"My first years of practice were materially lightened and helped forward by Dr. Fox, and I always felt when he left Portishead after having seen a case with me that he had in some way given my position another lift by his kindly expressions of approval to my patient's friends. He always seemed desirous of rather impressing the relations with the good I was doing than with the benefit that might accrue from his consultation.

"Since those days we have often met, and the same kindly voice has always called a greeting. Latterly we have been associated in many questions concerning the work of the three counties' branch of the National Association for the Prevention of Consumption, and the interest he from the first took in this movement has indeed been of great help, and has undoubtedly influenced general interest with its objects.

"A kinder, truer friend never lived, and the sorrow felt at his death is indeed deep and real by all who had the privilege of knowing him and his good work."

He lectured on medicine and pathological anatomy at the Bristol Medical School, in conjunction with Dr. Samuel Martin, from 1869 to 1874.

In 1870 he was elected a Fellow of the Royal College of Physicians of London, and in 1882 he delivered the Bradshaw Lecture.

At the foundation of the Clifton College in 1862 Dr. Fox was appointed to the office of Physician to the College, where at his suggestion Mr. Augustin Prichard was associated with him as his surgical colleague. He continued in this office up to the time of his resignation in 1882. He was much loved and valued by everyone connected with the school, where his kindness to the masters, more especially the younger ones, his unceasing attention to any case of severe illness arising amongst the boys, his prompt and unerring decisions, his constant presence at any function in the College, and his frequent attendance at the College Chapel, caused him to be one of the best known and respected of the College staff, amongst whom he has left so many loving friends.

When in the early sixties an epidemic of typhus fever broke out in one of the overcrowded and poorer districts of Bristol, Dr. Fox in conjunction with others established a temporary hospital for these cases, and did most excellent work in personally supervising the removal and after-care of many of the patients; for in those days systematic removal of the patients from their homes was a somewhat new departure, and many of them refused to go unless Dr. Fox went himself to persuade them.

He was a member of the Royal Medical and Chirurgical and Neurological Societies, and a Past-President of our own Bristol Medico-Chirurgical Society and of the Bath and Bristol Branch of the British Medical Association. When the Association met in Bristol in 1894 Dr. Fox was elected President, and much of the success of that meeting was due to his genial presence and popularity. His Presidential Address on "The Medical Man and the State," and his further address as President of the National Temperance League, attracted much attention at the time. His persistent advocacy of abstinence and his almost life-long practise of it enabled him to speak with much authority, and his words were cheered with great enthusiasm.

A colleague on the Council of the British Medical Association writes warmly of his work on behalf of this body: "In him the Association has lost a friend whom it can ill spare. In its early years, when the Association was encompassed by debts and a cloud of difficulties of all kinds and on all hands, Dr. Long Fox was better known amongst the rank and file of its members than he was in later years." He did much to help it through the difficulties of those early years, and "those who knew him longest will most keenly feel the loss the Association has sustained."

Of his many contributions to medical literature, of which we append a summary, the more noteworthy are the two larger works, *The Influence of the Sympathetic on Disease* and *The Patho-*

*logical Anatomy of the Nervous Centres.* Both of these were at the time regarded as standard works on the nervous system, giving evidence of much original thought and careful research. In 1881 he gave the Presidential Address to the Bristol Medico-Chirurgical Society, and chose for his subject "The Medulla Oblongata."

The local branch of the National Association for the Prevention of Consumption was much indebted to him in acting as Chairman of the General Committee from its initiation till within a few months of his death. No one took a keener interest in the promotion of this work, and it was at a meeting in Dr. Long Fox's drawing-room at Church House that it was decided to take some action towards the establishment of a local branch and towards the establishment of the Three Counties' Sanatorium which it is now proposed to erect at Winsley for the counties of Gloucester, Somerset and Wilts.

In middle life Dr. Fox was frequently the subject of acute attacks of gout, but for many years he remained almost free from acute symptoms. About eight years ago he suffered from pains in the lumbar region and some polyuria, which led him to test for and discover the existence of glycosuria. But with characteristic pluck and determination he continued his practice with undiminished devotion, and was enabled to carry on his work up to his last and fatal illness. In July of last year he went to West Malvern, hoping much from rest and change of air. He was able to resume work on his return for a short time only, when his increasing weakness compelled him with great reluctance to desist. He suffered acute pain for many weeks, associated with a symmetrical multiple neuritis, affecting the forearms and legs. On Christmas eve thrombosis of the right femoral vein occurred. This cleared up; but later, a few weeks before his death, thrombosis of the left femoral vein supervened. Towards the end his heart became dilated, and a well-marked mitral bruit was audible. Throughout his illness and during the last few months when Dr. Shaw and his son-in-law, Dr. Watson Williams, were in constant attendance, he always exhibited the greatest patience in his suffering and the tenderest solicitude for those around him.

The funeral service was held at Clifton Parish Church on March 31st, when the presence of a very large number of mourners assembled to show their regard for the deceased testified how great a loss the city, and more especially the medical part of it, had sustained.

We have been so accustomed to see Dr. Fox always present at any medical function, that no meeting has seemed complete without him; his genial personality and his wise counsel have been so long looked for and so rarely sought in vain, that the gap caused by his death must long remain unfilled. Bristol has lost one of her best loved and most prominent citizens: the medical profession has lost one who has long figured as its head, and to whom the following lines may well be applied:—

[1] The wisdom of thy common sense,
Thy honest hate of vain pretence,
Thy love and wide benevolence
Full often lead thee
Where feeling is its own defence.

And well the poor man thou befriendest,
And oftentimes an ill amendest.

---

### BIBLIOGRAPHY OF THE WORK OF EDWARD LONG FOX.

"Traumatic Tetanus," *Brit. M. J* , 1860, i. 189.

"Delirium Tremens," *Ibid.*, 915.

"Licence of the College of Physicians" [Letter], *Med. Times & Gaz.*, 1861, ii. 598.

"Practical Difficulties in the Diagnosis of Acute Phthisis," *Brit. M. J.*, 1862 ii. 612.

"Rubeola Notha" [Letter], *Lancet*, 1864, ii. 84.

"The Bromides" [Letter], *Brit. M. J.*, 1866, ii. 175.

"Aphasia Associated with Right Hemiplegia," *Lancet*, 1866, ii. 145.

"On Acute Tubercle," *Brit. M. J.*, 1868, i. 426.

"The Relation of the Secretion of Phosphoric Acid to Temperature in Certain Conditions of the Nervous System," *Ibid.*, 1868, ii. 544.

"A Peculiar Case of Hæmatemesis," *Ibid.*, 1869, ii. 568.

"Clinical Observations on the Temperature of Disease," *Med. Times & Gaz.*, 1870, i. 15, 144, 266, 360, 490, 601; *Ibid.*, 1870, ii. 5, 149, 262, 388, 526, 638.

"Case of Acute Chorea—Cerebral Hæmorrhage, twelve hours before death—with Minute Vegetations along the Edge of the Mitral Valves, and Microscopic Embolism of Corpus Striatum," *Ibid.*, 1870, ii. 423.

"Hydatids of the Liver," *Brit. M. J.*, 1871, i. 499.

[1] "To Burns," *Monthly Review*, No. 18, March, 1902, p. 157.

"The Nosology and Treatment of Diarrhœa, Cholerine and Asiatic Cholera," *Ibid.*, 1871, ii. 381.

"Tuberculous Phthisis," *Ibid.*, 463.

"On Some Abnormal Conditions of the Liver, Accompanied by Jaundice," *Ibid.*, 1872, ii. 8.

"Certain Pathological Conditions of the Nervous System," *Ibid.*, 1873, ii. 9.

"On Some Recent Results in Pathological Histology, and on Scientific Therapeutics" [Presidential Address to the Bath and Bristol Branch of the British Medical Association], *Ibid.*, 222.

*The Pathological Anatomy of the Nervous Centres*, 8vo., viii. (il.), 401 pp., 19 pl., Smith, Elder & Co., London, 1874.

"Cerebro-Spinal Sclerosis, *Med. Times & Gaz.*, 1874, ii. 143.

"A Case of Bulbar Paralysis," *Brit. M. J.*, 1876, ii. 243, 589.

"Clinical Lecture on Spinal Hæmorrhage," *Med. Times & Gaz.*, 1876, ii. 219.

"Treatment of Sunstroke" [Letter], *Lancet*, 1876, ii. 444.

"Paralysis of the Diaphragm, with Peculiar Laryngeal Symptoms," *Brit. M. J.*, 1877, ii. 252.

"The Temperature in Phthisis and Tuberculosis," *Brit & Foreign M.-Chir. Rev.*, 1877, lx. 253.

"Contributions to the Pathology of Tetanus," *Tr. Bristol M.-Chir. Soc.*, 1878, i. 60.

"Debate on the Treatment of High Temperature of the Body," *Ibid.*, 138.

"Acute Myelitis, as Illustrating the Physiology of the Spinal Cord," *Bristol Roy. Infirmary Rep.*, 1878-79, i. 1.

"Case of Mr. Crosby Leonard," *Ibid.*, 210.

*The Medulla Oblongata* [Presidential Address to the Bristol Medico-Chirurgical Society], 8vo., 18 pp., 1 pl., J. W. Arrowsmith, Bristol, 1881.

"On the Relations of the Conditions of the Blood and Blood-Vessels to the Health of the Tissues" [Presidential Address to the Section of Medicine, Annual Meeting of the British Medical Association, Ryde, Isle of Wight], *Brit. M. J.*, 1881, ii 349.

"Note on the Curability of Tabes Dorsalis," *Lancet*, 1882, i. 5.

"The Bradshaw Lecture on the Influence of the Sympathetic on Disease," *Brit. M. J.*, 1882, ii. 343, 399; *Lancet*, 1882, ii. 303: *Med. Times & Gaz.*, 1882, ii. 261.

Articles in *A Dictionary of Medicine*, edited by Richard Quain, 1883.

"Two Cases of Compression of the Spinal Cord by Sarcomatous Growths from the Soft Membranes," *Bristol M.-Chir. J.*, 1883, i. 100.

"Case of Spontaneous Cure of Spina Bifida, followed by Hydrocephalus," *Ibid.*, 1884, ii. 45.

"The Nature and Treatment of Chorea," *Ibid.*, 73.

*The Influence of the Sympathetic on Disease*, 8vo., xii., 565 pp., 11 pl., Smith, Elder & Co., London, 1885.

"Mangana Water," *Bristol M.-Chir. J.*, 1885, iii. 47.

"The Action of Diuretics," *Brit. M. J.*, 1885, ii. 340.

"Enlargement of the Spleen," *Bristol M.-Chir. J.*, 1885, iii. 145.

"The Therapeutics of the Neuroses," *Brit. M. J.* [illegible] i. 772.

"Case of Dysphagia Accompanied by Ascites," [illegible]87, i. 105.

"Nerve Storms," *Lancet*, 1890, i. 345.

"The Hand as a Diagnostic Factor in Diseases of th[illegible] Nervous System," *Medical Annual*, Bristol, 1891, p. 54.

Article on "The Sympathetic Nervous System," in *A Dictionary of Psychological Medicine*, edited by D. Hack Tuke, vol. ii., 1[illegible]

"On the Medical Man and the State" [Presidential Address delivered at the Sixty-second Annual Meeting of the British Medical Association, Bristol], *Brit. M. J.*, 1894, ii. 237; *Lancet*, 1894, ii. 288.

"The Use of Alcohol from a Medical Standpoint" [Speech in Debate], *Bristol M.-Chir. J.*, 1896, xiv. 94.

"Why do not the Members of the Medical Profession help the Cause of Temperance more?" *Med. Pioneer*, Lond., 1897, v. 92.

Address to the National Temperance League at Oxford on "The Physical Advantages of Abstinence," *Med. Temperance Rev.*, 1898, i. 64.

For Biography, see also *Med. Pioneer*, Lond., 1893-4, ii. 177; also *Prov. M. J.*, Leicester, 1894, xiii. 337, portr.; also *Brit. M. J.*, 1902, i. 861, portr.; also *Lancet*, 1902, i. 997.

## Figures and Tables

**Figure f1:**